# Changes in root-associated fungal communities in *Triticum aestivum* ssp. *spelta* L. and *Triticum aestivum* ssp. *vulgare* L. under drought stress and in various soil processing

**DOI:** 10.1371/journal.pone.0240037

**Published:** 2020-10-06

**Authors:** Sylwia Salamon, Katarzyna Mikołajczak, Lidia Błaszczyk, Karolina Ratajczak, Hanna Sulewska

**Affiliations:** 1 Department of Pathogen Genetics and Plant Resistance, Institute of Plant Genetics, Polish Academy of Sciences, Poznan, Poland; 2 Department of Agronomy, Poznan University of Life Sciences, Poznan, Poland; Institute of Genetics and Developmental Biology Chinese Academy of Sciences, CHINA

## Abstract

Plant roots are inhabited by an enormous variety of microorganisms, including fungi, which can control the growth as well as regulate the health of the host plants. The mycobiome composition of the roots of wheat plants, especially spelt, under drought stress has been rarely investigated. Therefore, the aim of the present study was to examine the composition of fungal communities in the root endosphere and rhizosphere of three *Triticum aestivum* ssp. *spelta* L. cultivars and one *Triticum aestivum* ssp. *vulgare* L. cultivar, grown under drought and controlled conditions in different soil preparations. Culture-dependent fungal community profiling was performed to examine the impact of rhizocompartments (endosphere, rhizosphere), host genotype, watering status and different soil preparation on roots mycobiome structure. A total of 117 fungal strains, belonging to 22 genera, were found to colonize the internal and external parts of roots in *T*. *aestivum* ssp. *spelta* L. and *T*. *aestivum* ssp. *vulgare* L. cultivars. The results showed that the part of root and soil preparation type significantly determined the mycobiome composition of wheat roots.

## Introduction

Plant roots are inhabited by different types of microorganisms, including fungi, which can control the growth as well as regulate the health of the host plants. Fungi present in the soil ecosystem not only act as substantial decomposers of biomass and plant symbionts but also pose threats as serious pathogens. These microorganisms are capable of colonizing both the internal and external parts of the plant organs. Fungi that colonize the internal tissue of a plant (endosphere) throughout or at least a part of their life cycle without manifesting any disease in the host are called endophytes [1]. Some of these fungi are known to improve the host’s defense against abiotic and biotic stresses, promote its growth, and reduce/inhibit the expansion of pathogens [2, 3]. The rhizosphere is an external territory, which closely surrounds the plant roots. Plants attract selected fungi inhabiting the soil by producing the exudates, and as a result, the structure of fungal communities in the rhizosphere differs from that in the adjacent soil area [4, 5]. Among the fungal species known to colonize the rhizosphere, the arbuscular mycorrhizal fungi (AMF) are observed in more than 80% of the terrestrial plants [6]. The AMF establish a conjunction between their host and the soil by creating an external hyphal network, which enhances the fitness of the host plant, increases its nutrient uptake, and improves its productivity under drought stress [7]. Apart from the beneficial fungi, some pathogenic species also colonize the rhizosphere. The most detrimental fungal pathogens that inhabit wheat roots are *Gaeumannomyces graminis* and those belonging to the *Fusarium* genus (*F*. *avenaceum*, *F*. *culmorum*, *F*. *graminearum*, *F*. *oxysporum*, and *F*. *poae*) [8–10]. Wheat (*Triticum aestivum* ssp. *vulgare* L.) and spelt wheat (*Triticum aestivum* ssp. *spelta* L.) are considered as an important source of nourishment for humans and livestock. Therefore, wheat cultivation is extensively performed and is of substantial economic importance [11, 12]. Furthermore, spelt wheat grain is identified as a great source of fiber, vitamins (A, E, D), and microelements (selenium, zinc, copper), and thus, its consumption is known to have a positive impact on human health [13]. It is one of the oldest cereals cultivated during the Roman period, and has regained popularity over the last 30 years. The growing nutritional requirements caused by increasing human population indicate the need for improving the global wheat production. Nonetheless, wheat plants are facing enormous challenges from abiotic and biotic stresses, especially during unpredictable weather conditions arising from climate changes. Drought, salinity, increase or decrease in temperature (hot or cold environment), flooding, ultraviolet radiation, and metal toxicity are the most deleterious abiotic stresses, which reduce crop productivity by up to 50% [14]. Wheat is sensitive to drought stress, especially until heading or germination stage and during the grain filling period [15]. Numerous phenological, physiological, and biochemical changes that are detrimental to wheat plants occur following the period of water deficiency. Drought leads to around 50% increase in the root/shoot ratio, due to a higher level of abscisic acid, which together with auxin, cytokine, and gibberellic acid stimulates the growth of roots but represses the development of shoot [14, 16].

The purpose of this study was to examine the composition of fungal communities in the root endosphere and rhizosphere of three *T*. *aestivum* ssp. *spelta* L. cultivars and one *T*. *aestivum* ssp. *vulgare* L. cultivar, grown under drought and well-watered conditions, in different soil preparations. The study focused on finding answers for two main questions: 1) Does the fungal mycobiome structure vary depending on the area of wheat roots (external and internal), host genotype, watering status, and different soil preparation? 2) Does a specific pattern of root mycobiome occur during drought in wheat plants/do some fungal species or genera specifically colonize the roots of these plants under conditions of water deficiency? Knowledge about the structure and diversity of root mycobiome in wheat is not only important from the ecological point of view but also promising for application in agricultural studies. This study on the native endosphere- and rhizosphere-associated fungal communities of wheat plants, conducted using cultivation-based approaches, might help in the identification of beneficial plant fungi for use in modern agronomy to create new-generation natural growth stimulators or factors capable of increasing plant adaptability to environmental changes.

## Materials and methods

### Plant material and experimental design

The experimental analyses were performed on the roots of *T*. *aestivum* ssp. *spelta* L. and *T*. *aestivum* ssp. *vulgare* L. plants. Initially, the plants were grown on the experimental field in Złotniki Research Station (52°48′ N, 16°82′ E, Poland), belonging to the Research and Education Center of Gorzyń, Poznań University of Life Sciences. When the wheat seedlings grew up to 8 cm long, they were collected, rinsed in running water, and disinfected using potassium manganate (0.05% KMnO₄) for 1 min. Then, two seedlings found showing the best growth performance were transplanted to polyethylene plastic pots (volume: 7 L). The plants were grown in the greenhouse for 4 months (from April to July) at a temperature between 18°C and 30°C, under a photoperiod of 16:8 h and relative humidity of 60–70%. All the plants were regularly fertilized using Florovit (5 mL/2 L H_2_O) and ammonium nitrate (1 g/pot) 1 month after the beginning of the experiment.

**Fig 1:**
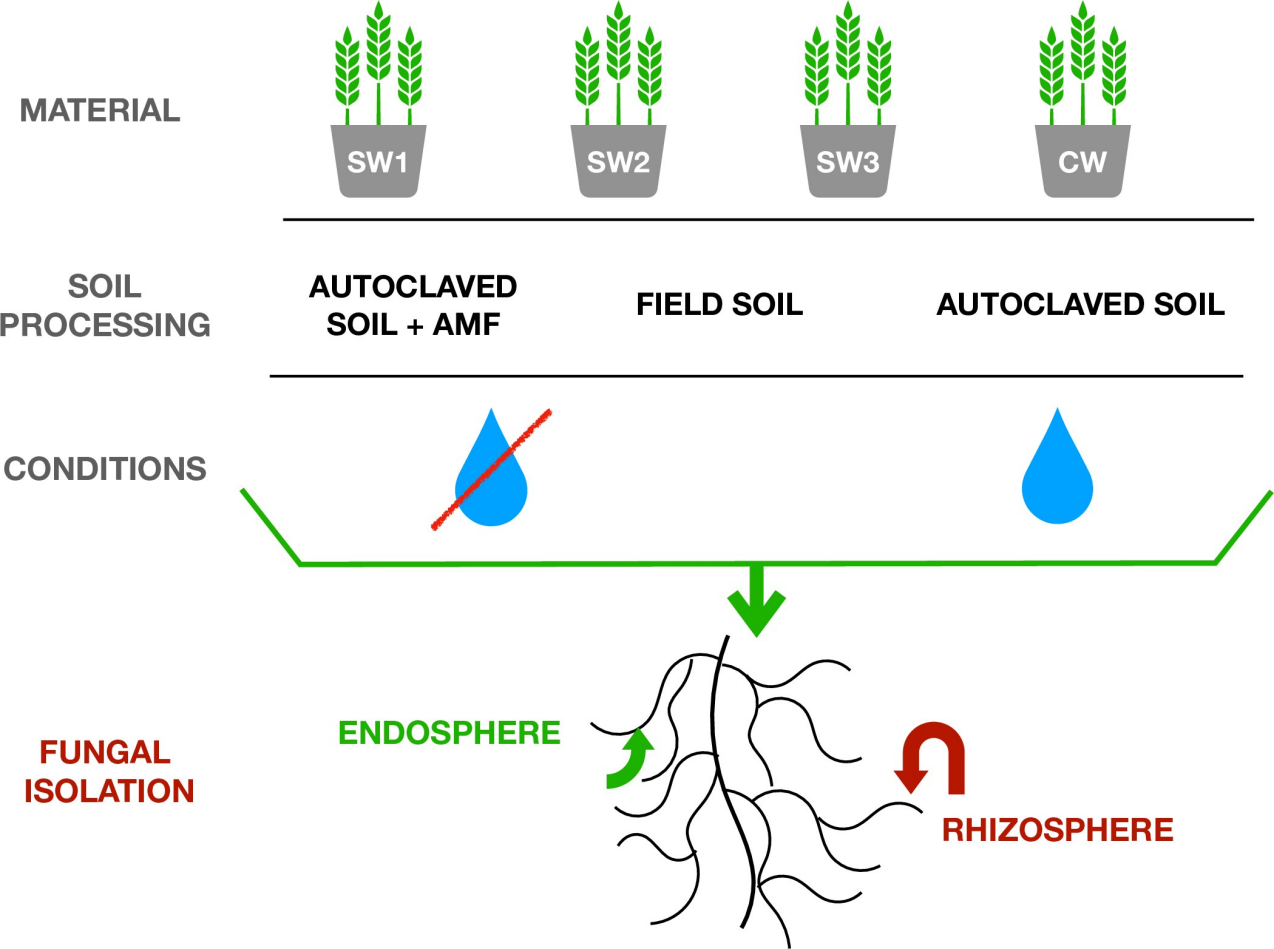
Research design of the conducted experiment. SW: spelt wheat, 1: ‘Badenstern’, 2: ‘Badenkrone’, 3: ‘Zollernspelz’; CW: common wheat ‘Dakotana’. AUTOCLAVED SOIL: control—sterile soil; FIELD SOIL: nonsterile soil, collected from Złotniki Research Station with a natural microbiological component; SOIL + AMF: autoclaved soil with the addition of arbuscular mycorrhizal fungi: DAOM 197198 strain of *Rhizophagus irregularis*.

The experimental design was completely randomized in a 2×4×3 factorial scheme with four replications (Fig 1): four wheat cultivars (common wheat: ‘Dakotana’ (KWS Saat, Einbeck, Germany); spelt wheat: ‘Badenstern’, ‘Badenkrone’ (both ZG Raiffeisen eG, Karlsruhe, Germany), and ‘Zollernspelz’ (Saaten Union, Isernhagen, Germany)), two levels of drought stress (control—pots were irrigated with deionized water; drought—pots were not irrigated in the flowering phase), three types of soil preparations (control—sterile, autoclaved soil; nonsterile soil—collected from Złotniki Research Station, with a natural microbiological component; autoclaved soil with the addition of AMF (DAOM 197198 strain of *Rhizophagus irregularis*, syn. *Glomus irregulare*; Connectis, Agronutrition, France)), and two areas of roots (endosphere and rhizosphere).

### Soil preparation

The soil (0–20 cm layer depth) used for the greenhouse experiment was collected at the Złotniki Research Station, from the field where spelt wheat and common wheat were grown. According to the FAO/WRB classification [17], the soil used in the study was a luvisol of light clay sand grade, shallowly deposited on a light clay belonging to a good rye complex. The pH was 5.7, and it had an average phosphorus content of 120.6 mg P_2_O_5_ kg^-1^ (very high) and an average potassium content of 122.5 mg K_2_O kg^-1^ (high) with 0.59% organic matter. A part of the collected soil was first sieved in 4-mm mesh, autoclaved at 120°C for 1 h, and subsequently stored for 2 days before it was used in the greenhouse. Half of the pots filled with this autoclaved soil medium were inoculated by watering 2 mL per two plants of spore suspension of *R*. *irregularis* DAOM 197198 strain (2×10^3^ spores mL^−1^ sterile water), while the remaining pots filled with the autoclaved soil medium were treated with 2 mL of sterile water.

### Drought stress conditions

Drought stress was initiated in the flowering phase (BBCH 65–69) and was maintained for 8 days. The drought symptoms were evaluated in tested plants by observing the leaves vigor and measuring the soil humidity using a probe (ThetaProbe, Eijkelkamp Penetrologger SN, Giesbeek, The Netherlands). Wheat plants showing rolled-up leaf blades, grown in different soil variants with a low moisture level (5–8%), were selected for further analyses.

### Sampling and fungi isolation

To evaluate the fungal communities, the roots were separated from wheat plants at late milk maturity stage in BBCH 73–77 phase, after 8 days of drought. The collected roots were placed in separate paper bags, transported to the laboratory, and stored for 24 h at 4°C until processing.

Fungal isolates were obtained from endosphere and rhizosphere of both wheat and spelt wheat roots. To isolate the endophytic fungi, the plant roots were sectioned into 4- to 5-cm-long pieces and were surface sterilized by treating with 70% ethanol and 0.5% active chlorine and rinsing five times in sterile distilled water. Then, the root samples were cut with a sterile scalpel to obtain 1-cm-long sections. Both sterilized and nonsterilized parts of roots were placed on Potato Dextrose Agar (PDA; Oxoid™, Thermo Fisher Scientific, Waltham, Massachusetts, United States) supplemented with ampicillin (50 mg mL^-1^). The samples were incubated at 22°C for 1–2 weeks or until the emergence of mycelia. Putative fungal colonies were isolated by performing three (or more if needed) rounds of subculture on PDA, until a visually homogeneous culture (confirmed by observing under a light microscope (Zeiss)) was obtained. Pure cultures were either subsequently used for DNA isolation or were transferred to tubes containing SNA (synthetic nutrient-poor agar) and preserved in sterile mineral oil at 4°C for further analyses.

### DNA isolation, PCR amplification, and sequencing

For DNA isolation, 40 mg of mycelium obtained from homogeneous cultures was used. DNA was extracted from the mycelium using Wizard® Genomic DNA Purification Kit (Promega, Madison, Wisconsin, United States), according to the manufacturer’s protocol. For fungal identification, DNA sequencing of the internal transcribed spacer (ITS) region, small-subunit (SSU) or large-subunit (LSU) nrRNA, and protein-coding markers (beta-tubulin (*tub2*) and translation elongation factor 1-alpha (*tef1*)) was performed. The primers and PCR conditions used in the study are described in Table 1. DNA amplification was performed on a T-1000 Thermal Cycler (BioRad, Hercules, California, United States). Each PCR mix contained approximately 50 ng of DNA, 1.0 μM of each primer, 0.2 mM of each dNTP (Sigma-Aldrich, Saint Louis, Missouri, United States), and 1.25 unit of DreamTaq Green DNA Polymerase (Thermo Fisher Scientific, Waltham, Massachusetts, United States). After amplification, the PCR products were separated and examined on a 1.5% agarose gel added with Simply Safe dye (EURx, Poland) and 100-bp DNA Ladder Plus (Thermo Fisher Scientific, Waltham, Massachusetts, United States). Then, 2.5 μL of each amplicon was purified by incubating for 30 min at 37°C with 2 units of Exonuclease I (Thermo Fisher Scientific, Waltham, Massachusetts, United States) and 0.4 unit of FastAP Thermosensitive Alkaline Phosphatase I (Thermo Fisher Scientific, Waltham, Massachusetts, United States). Subsequently, the enzymes were inactivated at 80°C for 15 min. DNA sequencing was performed using the BigDye3.1® Kit under the following conditions: 96°C for 2 min; 25 cycles of 30 s at 96°C, 30 s at 50°C, and 4 min at 60°C. The sequencing product was cleaned using 0.4 g of Sephadex® G-50 beads (Sigma-Aldrich, Saint Louis, Missouri, United States), with a diameter of 20–50 μm, on MultiScreen Filter Plates HTS (Merck Millipore, Burlington, Massachusetts, United States). Capillary electrophoresis procedure was performed using ABI3730 Genetic Analyzer (Applied Biosystems, Foster City, California, United States) in the Institute of Biochemistry and Biophysics of the Polish Academy of Sciences (Warsaw).

**Table 1:** PCR primers and conditions applied for molecular fungal identification.

Locus	Amplicon length	Primer	Primer sequence 5’-3’	PCR conditions	Reference
**Internal Transcribed Spacer (ITS) region of the rRNA**	~450-800bp	ITS1F	CTT GGT CAT TTA GAG GAA GTA A	1. 95˚C– 5 min 2. 95˚C– 30 s 3. 52˚C– 30 s 4. 72˚C– 1 min Steps 2–4 x 35 cycles 5. 72˚C– 8 min	[18]
		ITS4	TCC TCC GCT TAT TGA TAT GC		
**Translation elongation factor 1-alpha (*tef1*)**	~600 bp	EF1-1018F	GAY TTC ATC AAG AAC ATG AT	1. 95˚C– 2 min 2. 66˚C-56˚C touchdown (9 cycles) 3. 95˚C– 30 s 4. 56˚C– 1 min 5. 72˚C– 1 min Steps 3–5 x 36 cycles 6. 72˚C– 10 min	[19]
		EF1-1620R	GAC GTT GAA DCC RAC RTT GTC		
**beta-tubulin (*tub2*)**	~ 500 bp	Bt2a	GGT AAC CAA ATC GGT GCT GCT TTC	1. 95˚C– 3 min 2. 95˚C– 30 s 3. 58˚C– 30 s 4. 72˚C– 1 min Steps 2–4 x 35 cycles 5. 72˚C– 10 min	[20]
		Bt2b	ACC CTC AGT GTA GTG ACC CTT GGC		
**Translation elongation factor 1-alpha (*tef1*)**	~700 bp	ef1	ATG GGT AAG GA(A/G) GAC AAG AC	1. 95˚C– 3 min 2. 95˚C– 30 s 3. 58˚C– 30s 4. 72˚C– 1 min Steps 2–4 x 35 cycles 5. 72˚C– 10 min	[21]
		ef2	GGA (G/A)GT ACC AGT (G/C)AT CAT GTT		
**Translation elongation factor 1-alpha (*tef1*)**	~700 bp	Ef728M	CATCGAGAAGTTCGAGAAGG	1. 95˚C– 3 min 2. 95˚C– 30 s 3. 58˚C– 30 s 4. 72˚C– 1 min Steps 2–4 x 35 cycles 5. 72˚C– 10 min	[22, 23]
		Tef1R	GCCATCCTTGGAGATACCAGC		
**Small Subunit (SSU, 18S) of the rRNA**	~1200 bp	NS1	GTA GTC ATA TGC TTG TCT C	1. 95˚C– 5 min 2. 95˚C– 30 s 3. 52˚C– 30 s 4. 72˚C– 1 min Steps 2–4 x 35 cycles 5. 72˚C– 8 min	[18]
		NS4	CTT CCG TCA ATT CCT TTA AG		
**Large Subunit (LSU, 28S) of the rRNA**	~1200 bp	LROR	ACC CGC TGA ACT TAA GC	1. 95˚C– 5 min 2. 95˚C– 30 s 3. 52˚C– 30 s 4. 72˚C– 1 min Steps 2–4 x 35 cycles 5. 72˚C– 8 min	[24, 25]

### Bioinformatics and statistical analysis

The DNA sequences were analyzed using DNA Star Software (Madison, Wisconsin, United States) and BLASTn (Basic Local Alignment Search Tool) algorithm (National Centre for Biotechnology Information (NCBI), http://www.ncbi.nlm.nih.gov/BLAST/). All the sequences contained fragments of ITS, SSU and LSU regions, and *tub2* and *tef1* genes. These were deposited in NCBI GenBank (https://www.ncbi.nlm.nih.gov/genbank/), and their accession numbers are provided in S1 Table. Charts illustrating the composition of fungal communities identified in the tested conditions were prepared using ggplot2 and VennDiagram packages for R software [26, 27]. Evolutionary analyses were conducted in MEGA X [28], by applying the Maximum Likelihood method and the Tamura–Nei model [29]. The bootstrap consensus tree inferred from 500 replicates was taken to represent the evolutionary history of the sequences analysed [30]. The initial trees for the heuristic search were obtained automatically by applying the Neighbor-Joining and BioNJ algorithm to a matrix of pairwise distances estimated using the Maximum Composite Likelihood approach, and then selecting the topology that had the superior log-likelihood value. A discrete Gamma distribution was applied to model the evolutionary rate differences among sites (5 categories (+G, parameter = 0.8233)). Furthermore, the rate variation model was used to allow some sites to be evolutionarily invariable ([+I], 14.86% sites). The phylogeny tree was generated using the iTOL software, v. 5.5.1 [31].

To examine the differences in the structure of fungal communities among the studied groups, we performed principal coordinate analysis (PCoA), based on the unweighted and weighted UniFrac distances, using phyloseq package in R [32]. To determine the significance of differences observed in the calculated distances, permutational multivariate analysis of variance (PERMANOVA) with a permutational number of 999 was applied, using the *adonis* function of the vegan package in R [33].

## Results

**Fig 2:**
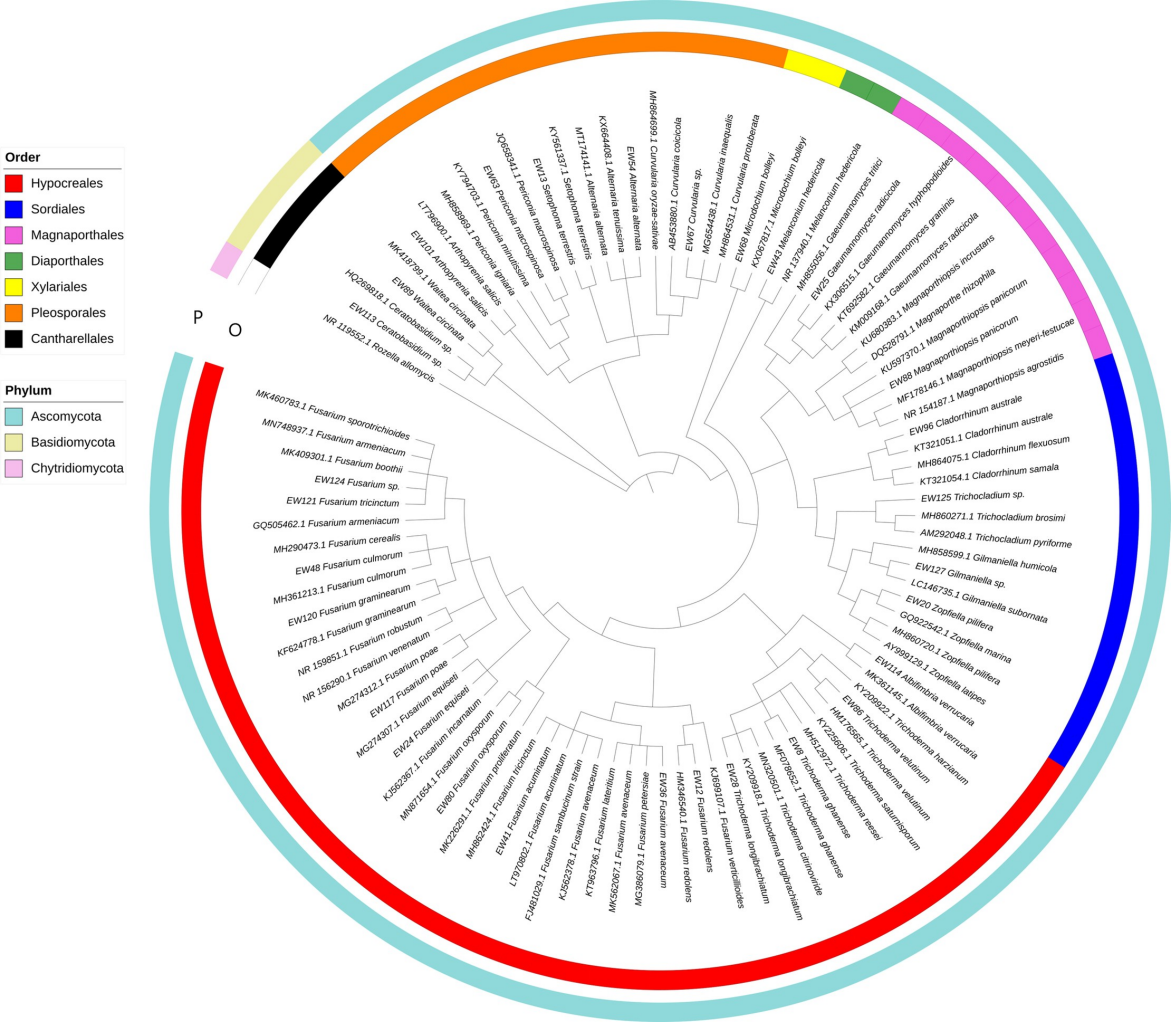
Evolutionary analysis of the ITS region of root-associated fungi identified in common and spelt wheat, using the Maximum Likelihood method.

A total of 117 fungal strains were isolated from the endosphere and rhizosphere of the common wheat cultivar ‘Dakotana’ and the spelt wheat cultivars ‘Badenstern’, ‘Badenkrone, and ‘Zollernspelz’, grown in three types of soil preparations (control—autoclaved, sterile soil; nonsterile soil—collected from Złotniki Research Station, with a natural microbiological component; autoclaved soil with AMF (DAOM 197198 strain of *R*. *irregularis*) added), under two different growth conditions (watered and not watered during the flowering phase). The majority of the identified fungi belonged to phylum *Ascomycota*, and among them, the orders *Hypocreales* and *Pleosporales* were found to be predominant (Fig 2). *Waitea circinata*, *Ceratobasidium* sp., *Marasmius* sp., and *Rhizoctonia solani* were the only *Basidiomycota* observed in the studied wheat roots. A higher number of fungal strains were observed in the rhizosphere than in the endosphere (63 and 54 strains, respectively); however, the endophytic fungal communities demonstrated a similar level of diversity (25 and 27 strains were observed, respectively). Of the identified fungi, 33% were present in both the internal and external parts of the roots. Nonetheless, *Albifimbria verrucaria*, *Alternaria alternata*, *Curvularia* sp., *Fusarium acuminatum*, *F*. *culmorum*, *Fusarium equiseti*, *F*. *poae*, *Gaeumannomyces radicicola*, *Melanconium hedericola*, *Mucor circinelloides*, *Marasmius* sp., *Rhizoctonia* sp., *Trichoderma velutinum*, and *Trichoderma ghanense* were observed only in the rhizosphere and not in the endosphere, whereas *Arthopyrenia salicis*, *Gilmaniella* sp., *F*. *graminearum*, *Magnaporthiopsis panicorum*, *Magnaporthiopsis* sp., *Penicillium crustosum*, *Periconia macrospinosa*, *Trichocladium* sp., *Trichoderma* sp., *Setophoma* sp., *Zopfiella pilifera*, and *Zopfiella* sp. were observed only in the inner part of the plant roots.

The analysis involved 95 nucleotide sequences and contained a total of 695 positions in the final dataset. Branches corresponding to partitions reproduced in less than 50% of bootstrap replicates (500 replicates) are collapsed.

**Fig 3:**
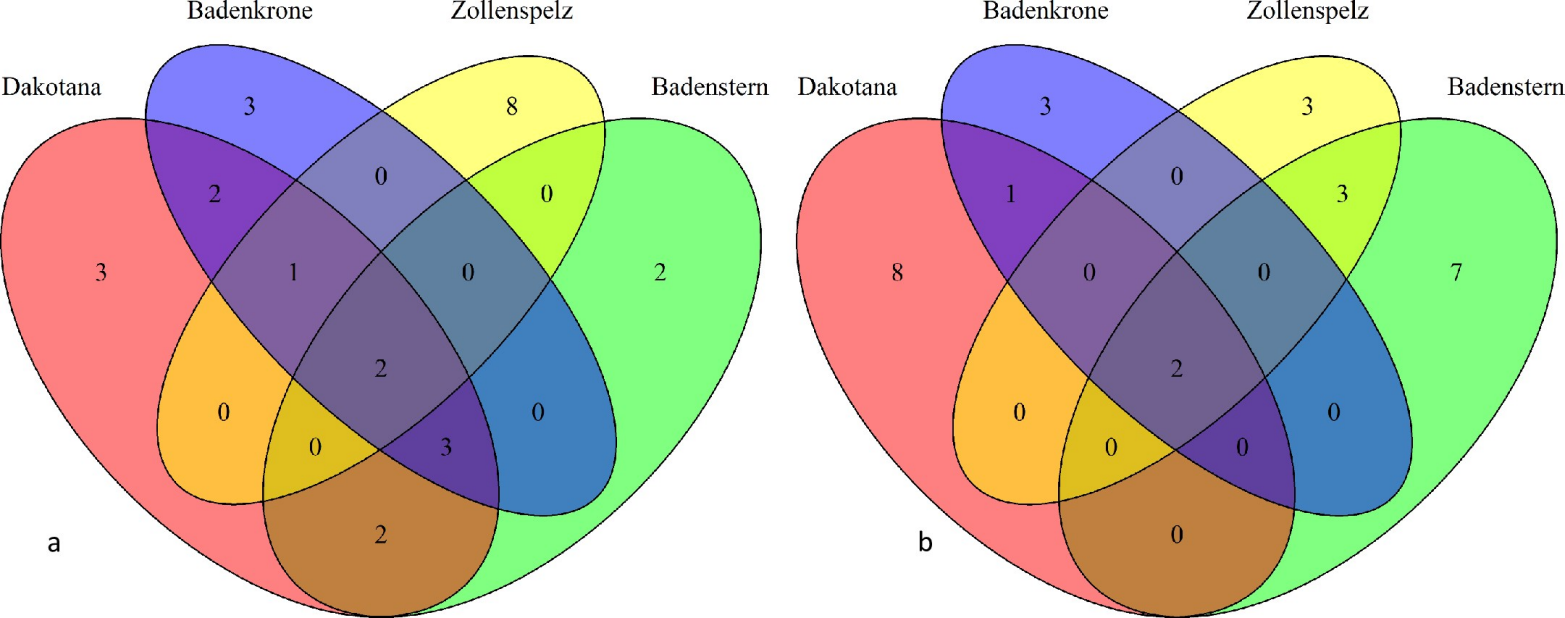
Comparison of fungi identified in ‘Badenstern’, ‘Badenkrone, ‘Zollernspelz’, and ‘Dakotana’ wheat in the root (a) endosphere and (b) rhizosphere.

The composition of fungal communities was also found to vary depending on the host genotype (Fig 3). In the endosphere, only *Microdochium bolleyi* and *Setophoma terrestris* were identified in all the studied cultivars. The ‘Zollernspelz’ cultivar demonstrated the most unique pattern of fungal composition in the root endosphere. By contrast, 65% of the fungi observed in the rhizosphere of this cultivar were similar to those observed in the ‘Badenstern’ cultivar. *Fusarium avenaceum* and *Fusarium redolens* were the only fungal species observed in the rhizosphere of all the wheat genotypes analyzed in the study. A majority of the fungi identified in the rhizosphere of ‘Dakotana’ were found to be exclusive to this cultivar, whereas 75% of the fungal endophytes observed in this cultivar were also detected in the remaining analysed wheat genotypes.

In general, the composition and abundance of the wheat root-associated fungi were not found to be significantly influenced by drought stress. Fungi identified in wheat roots under drought and control conditions, were listed in Table 2. *Fusarium avenaceum*, *M*. *bolleyi*, *Cladorrhinum australe*, *F*. *redolens*, *W*. *circinata*, *F*. *oxysporum*, *Ceratobasidium* sp., *R*. *solani*, *T*. *ghanense*, *Fusarium tricinctum*, *Periconia* sp., and *S*. *terrestris* were observed in both plants grown under drought and those grown in well-watered conditions. However, *Trichoderma longibrachiatum* and *T*. *velutinum* were observed only in the plants grown under drought stress, while *Zopfiella* sp., *M*. *hedericola*, *A*. *verrucaria*, *G*. *radicicola*, and *A*. *salicis* were observed exclusively in the irrigated plant groups.

**Table 2:** Comparison of fungi identified in roots endosphere and rhizosphere of wheat plants under drought and controlled conditions.

	ENDOSPHERE		RHIZOSPHERE	
	DROUGHT	CONTROL	DROUGHT	CONTROL
**1.**	*Cladorrhinum australe*	*Arthopyrenia salicis*	*Alternaria alternata*	*Albifimbria verrucaria*
**2.**	*Fusarium avenaceum*	*Ceratobasidium sp*.	*Ceratobasidium sp*.	*Cladorrhinum australe*
**3.**	*Fusarium oxysporum*	*Cladorrhinum australe*	*Cladorrhinum australe*	*Culvularia sp*.
**4.**	*Fusarium redolens*	*Fusarium avenaceum*	*Fusarium avenaceum*	*Fusarium acuminatum*
**5.**	*Fusarium sp*.	*Fusarium graminearum*	*Fusarium culmorum*	*Fusarium avenaceum*
**6.**	*Gilmaniella sp*.	*Fusarium oxysporum*	*Fusarium redolens*	*Fusarium equiseti*
**7.**	*Magnaporthiopsis panicorum*	*Fusarium redolens*	*Fusarium sp*.	*Fusarium poae*
**8.**	*Microdochium bolleyi*	*Fusarium sp*.	*Fusarium tricinctum*	*Fusarium redolens*
**9.**	*Penicillium crustosum*	*Fusarium tricinctum*	*Microdochium bolleyi*	*Gaeumannomyces radicicola*
**10.**	*Periconia macrospinosa*	*Magnaporthiopsis sp*.	*Periconia sp*.	*Marasmius sp*.
**11.**	*Rhizoctonia solani*	*Microdochium bolleyi*	*Rhizoctonia sp*.	*Melanconium hedericola*
**12.**	*Setophoma terrestris*	*Periconia sp*.	*Setophoma terrestris*	*Microdochium bolleyi*
**13.**	*Trichocladium sp*.	*Setophoma sp*.	*Trichoderma ghanense*	*Mucor circinelloides*
**14.**	*Trichoderma longibrachiatum*	*Setophoma terrestris*	*Trichoderma longibrachiatum*	*Rhizoctonia solani*
**15.**	*Waitea circinata*	*Trichoderma sp*.	*Trichoderma velutinum*	*Setophoma terrestris*
**16.**	-	*Waitea circinata*	*Waitea circinata*	*Trichoderma ghanense*
**17.**	-	*Zopfiella pilifera*	-	*Waitea circinata*
**18.**	-	*Zopfiella sp*.	-	-

Soil preparation strongly affected the mycobiome structure of wheat roots. Table 3 shows fungi identified in wheat roots grew in soils after three different types of processing. The roots collected from the plants grown in nonautoclaved field soil demonstrated the most diverse mycobiome composition (21 taxons). Moreover, more than 70% of the identified fungi (*A*. *salicis*, *Curvularia* sp., *Periconia* sp., *P*. *macrospinosa*, *Zopfiella* sp., *Z*. *pilifera*, *F*. *equiseti*, *Marasmius* sp., *Setophoma* sp., S. *terrestris*, *G*. *radicicola*, *M*. *circinelloides*, *Magnaporthiopsis* sp., *M*. *panicorum*, and *T*. *velutinum*) were observed exclusively in the nonautoclaved soil group. Fungi belonging to genera *Setophoma*, *Periconia*, *Magnaporthiopsis*, *Fusarium*, *Michrodochium*, and *Waitea* were most frequently isolated from the plants grown in nonautoclaved soil. However, wheat plants grown in the autoclaved soil also demonstrated equally high species richness (19 taxons), with almost 50% of the species identified were noticed only in this group (*A*. *alternata*, *F*. *culmorum*, *F*. *graminearum*, *F*. *tricinctum*, *Trichocladium* sp., *Trichoderma* sp., *T*. *longibrachiatum*, *Gilmaniella* sp., *Rhizoctonia* sp.). Only three of the observed species, namely *F*. *avenaceum*, *M*. *bolleyi*, and *W*. *circinata*, were commonly observed in all the analyzed soil preparations. Of the soil groups analyzed, the autoclaved and AMF-added groups showed high similarity in the structure of fungal communities (*A*. *verrucaria*, *Ceratobasidium* sp., *C*. *australe*, *F*. *poae*, *R*. *solani*, *T*. *ghanense*). By contrast, the field soil having a natural microorganism composition presented a diverse fungal reservoir; thus, only two and one species, belonging to genus *Fusarium*, were shared between the field soil and AMF-added soil and between the field soil and autoclaved soil, respectively.

**Table 3:** Fungi identified in roots endosphere and rhizosphere of wheat plants grew in soil after different preparation: Autoclaved (CONTROL), autoclaved with the addition of arbuscular mycorrhizal fungi: DAOM 197198 strain of *Rhizophagus irregularis* (AMF) and nonsterile collected on field (FIELD SOIL).

CONTROL	AMF	FIELD SOIL
*Albifimbria verrucaria*	*Albifimbria verrucaria*	*Arthopyrenia salicis*
*Alternaria alternata*	*Ceratobasidium sp*.	*Fusarium oxysporum*
*Ceratobasidium sp*.	*Cladorrhinum australe*	*Microdochium bolleyi*
*Cladorrhinum australe*	*Fusarium acuminatum*	*Curvularia sp*.
*Fusarium avenaceum*	*Fusarium avenaceum*	*Fusarium avenaceum*
*Fusarium culmorum*	*Fusarium oxysporum*	*Fusarium equiseti*
*Fusarium graminearum*	*Fusarium poae*	*Fusarium oxysporum*
*Fusarium poae*	*Fusarium redolens*	*Fusarium redolens*
*Fusarium sp*.	*Melanconium hedericola*	*Fusarium sp*.
*Fusarium tricinctum*	*Microdochium bolleyi*	*Gaeumannomyces radicicola*
*Gilmaniella sp*.	*Penicillium crustosum*	*Magnaporthiopsis panicorum*
*Microdochium bolleyi*	*Rhizoctonia solani*	*Magnaporthiopsis sp*.
*Rhizoctonia solani*	*Trichoderma ghanense*	*Microdochium bolleyi*
*Rhizoctonia sp*.	*Waitea circinata*	*Mucor circinelloides*
*Trichocladium sp*.		*Periconia macrospinosa*
*Trichoderma ghanense*		*Periconia sp*.
*Trichoderma longibrachiatum*		*Setophoma terrestris*
*Trichoderma sp*.		*Trichoderma velutinum*
*Waitea circinata*		*Waitea circinata*
		*Zopfiella pilifera*
		*Zopfiella sp*.

**Fig 4:**
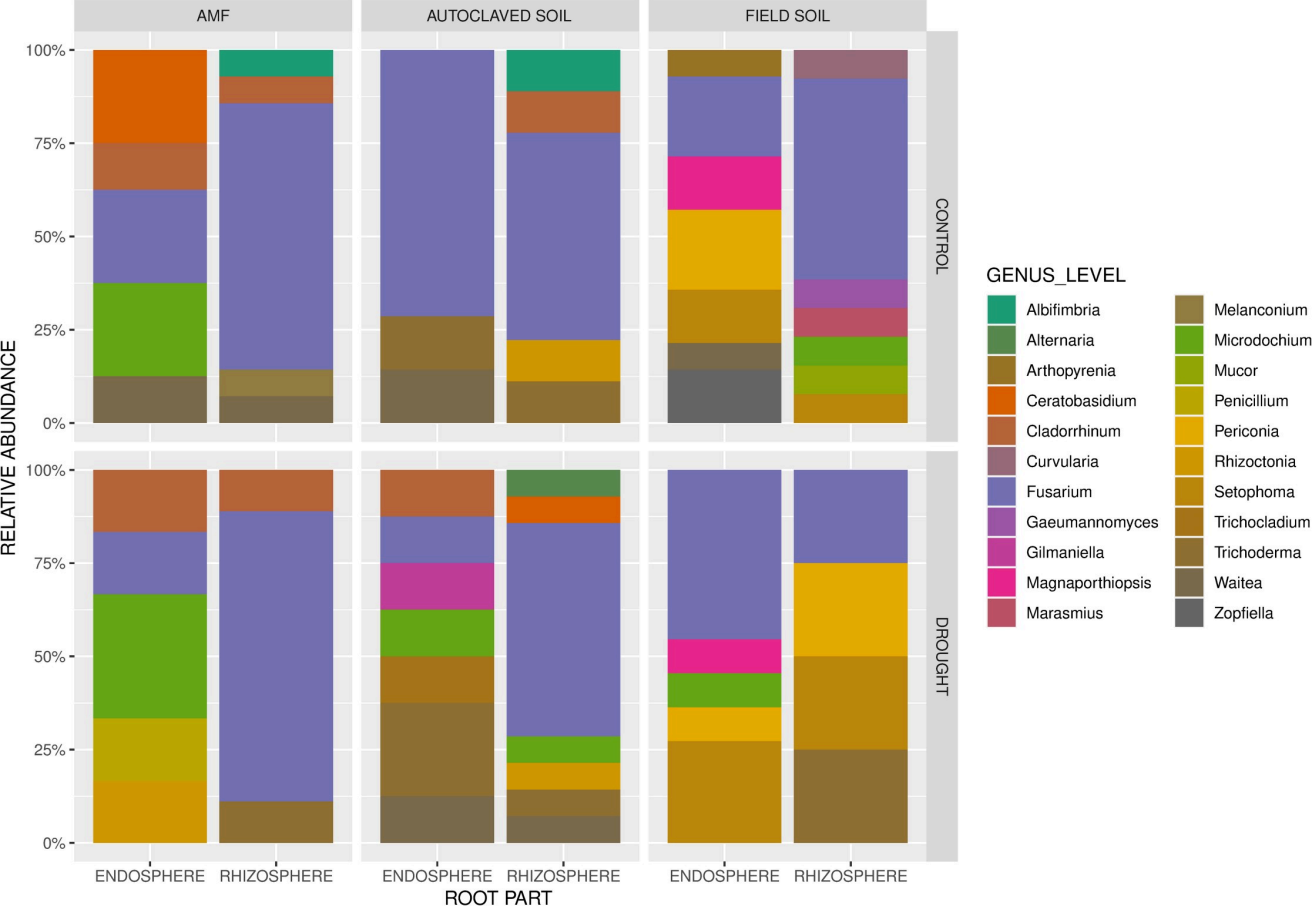
Genus-level relative abundance (%) of the identified fungi across different soil treatment, root part, and watering status.

The compiled results of fungal identification at the genus level across soil with different microbiological components, root parts, and watering status are shown in Fig 4. A distinct endophytic structure was observed in three studied soil preparation types depending on the drought existence. Greater number of different genera were identified in roots from field soil and from AMF soil under controlled condition, than in the same groups under drought stress. On the other hand, the opposite pattern was observed in the autoclaved soil, in which the fungal communities were found to be much more diverse in wheat roots under the water-deficient condition. The amount of *Fusarium* sp. was lower in the endosphere of plants grown in the AMF and autoclaved soil under drought, in contrast to the field soil plants in which the amount of these species was higher in the endosphere than in the rhizosphere. Moreover, *Trichoderma* sp. were found more often under drought, compared to the roots of wheat plants grown in controlled conditions.

**Fig 5:**
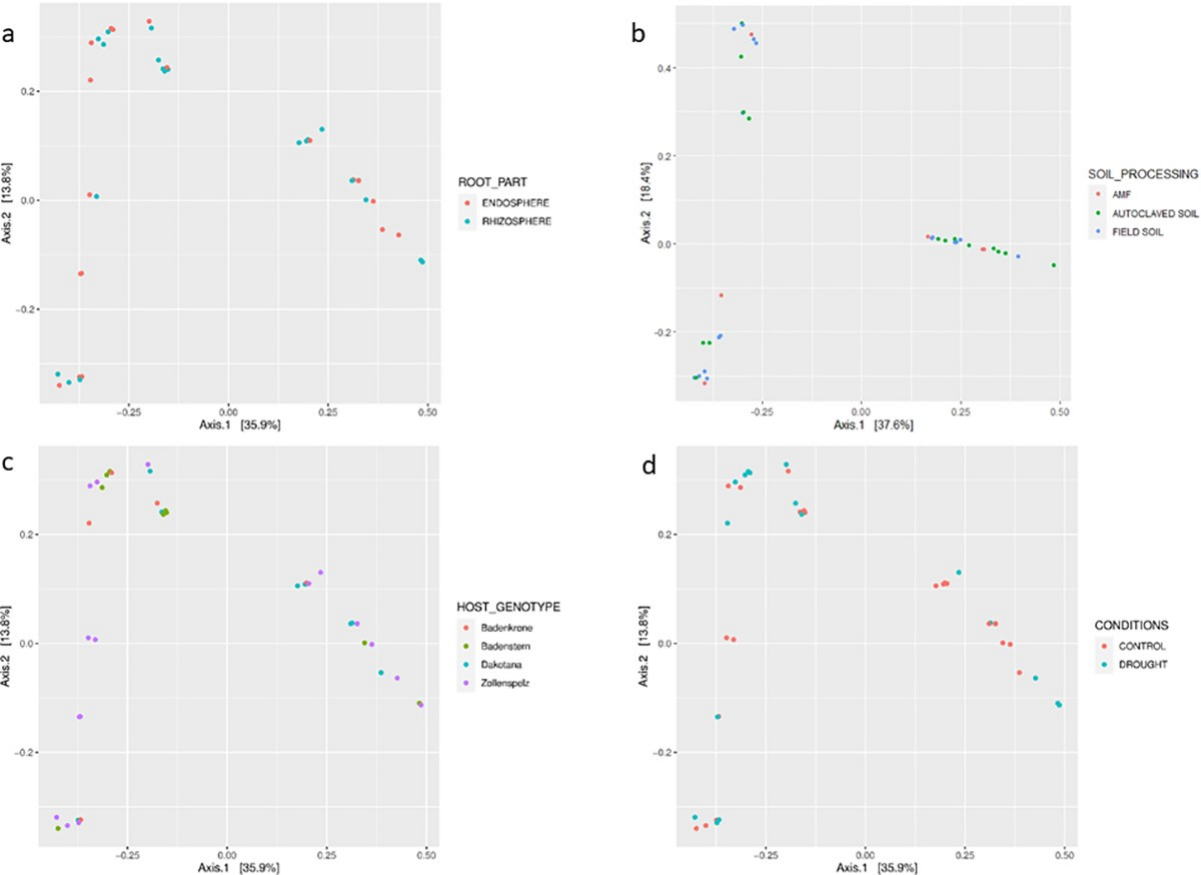
Principal coordinate analysis of the fungal community structure across different root parts, watering conditions, cultivars, and soil preparation with various microbiological components, based on unweighted UniFrac distances.

PCoA, performed based on unweighted (Fig 5) and weighted (data not shown) UniFrac distances, showed the differences in biological communities across parts of roots, watering conditions, host varieties, and different soil preparation. Unweighted UniFrac considers only the sequence distances, whereas weighted UniFrac also includes abundance information. PERMANOVA performed based on unweighted UniFrac distances revealed that the part of the roots and different soil treatment had a significant impact on the composition of fungal communities in wheat roots (*p*<0.05 and *p*<0.01, respectively). The influence of different soil preparation was also confirmed by PERMANOVA conducted based on weighted UniFrac (*p*<0.01).

## Discussion

Here, wheat plant have been used as a model to investigate the impact of rhizocompartments (endosphere, rhizosphere), host genotype, watering status and different soil preparation on roots mycobiome structure by detailed characterization of the root fungal communities. In the present study, we have demonstrated the existence of 20 species (*C*. *australe*, *F*. *avenaceum*, *F*. *equiseti*, *F*. *oxysporum*, *F*. *redolens*, *F*. *tricinctum*, *Fusarium* sp., *G*. *radicicola*, *Magnaporthiopsis* sp., *Marasmius* sp., *M*. *bolleyi*, *M*. *circinelloides*, *P*. *crustosum*, *Periconia* sp., *Setophoma* sp., *S*. *terrestris*, *T*. *ghanense*, *T*. *longibrachiatum*, *W*. *circinata*, *Z*. *pilifera*) in the internal parts and on the surface of the roots in common wheat and 31 species (*A*. *verrucaria*, *A*. *alternata*, *A*. *salicis*, *Ceratobasidium* sp., *C*. *australe*, *Curvularia* sp., *F*. *acuminatum*, *F*. *avenaceum*, *F*. *culmorum*, *F*. *graminearum*, *F*. *oxysporum*, *F*. *poae*, *F*. *redolens*, *F*. *tricinctum*, *Fusarium* sp., *Gilmaniella* sp., *M*. *panicorum*, *Magnaporthiopsis* sp., *M*. *hedericola*, *M*. *bolleyi*, *P*. *macrospinosa*, *Periconia* sp., *R*. *solani*, *Rhizoctonia* sp., *S*. *terrestris*, *Trichocladium* sp., *T*. *longibrachiatum*, *Trichoderma* sp., *T*. *velutinum*, *W*. *circinata*, *Zopfiella* sp.) in the case of spelt wheat. Most of the species identified in this study as inhabiting the roots of common wheat have already been described as wheat root-associated fungi in the previous studies [34–38]. Nevertheless, in the case of spelt wheat, knowledge on root-inhabiting fungal species remains limited [39].

We tracked the changes in roots mycobiome induced by applied conditions, and found out the impact of rhizocompartments and different soil preparation on fungal composition in wheat roots (Fig 5). We observed some compartment specific taxa: *A*. *verrucaria*, *A*. *alternata*, *Curvularia* sp., *F*. *acuminatum*, *F*. *culmorum*, *F*. *equiseti*, *F*. *poae*, *G*. *radicicola*, *M*. *hedericola*, *M*. *circinelloides*, *Marasmius* sp., *Rhizoctonia* sp., *T*. *velutinum*, and *Trichoderma ghanense* exclusively in the rhizosphere and *A*. *salicis*, *Gilmaniella* sp., *F*. *graminearum*, *M*. *panicorum*, *Magnaporthiopsis* sp., *P*. *crustosum*, *P*. *macrospinosa*, *Trichocladium* sp., *Trichoderma* sp., *Setophoma* sp., *Z*. *pilifera*, and *Zopfiella* sp. only in endosphere. A number of previous studies have focused on the endophytes of the common wheat—*T*. *aestivum* ssp. *vulgare* L.; however, they mainly analyzed the differences in structure between plant organs and management strategies or growth stages [35, 40–42]. To the best of our knowledge, no studies published thus far have investigated the fungal structure and dynamics of the endosphere and/or rhizosphere of spelt wheat—*T*. *aestivum* ssp. *spelta* L. However, the endogenous bacterial communities in the endosperm, germ, roots, coleoptiles, and leaves of spelt wheat were described in a recent work [43].

The type of the soil preparation also significantly influenced the mycobiome composition of wheat roots. Mycobiome structure comparison between roots from field soil and autoclaved soil (control) revealed that numerous of identified fungi occurred only in ‘field soil’ samples, this indicates origin from adjacent soil area. We assume that fungi observed in rhizosphere of roots grown in autoclaved soil derived from the inner part of the plant and was able to colonize the sterile niche. Furthermore, the mycobiome structure of roots from autoclaved (control) and AMF soil were similar, so addition of *Rhizophagus irregularis* didn’t have notable effect on structure of root-associated fungal communities in wheat. The root-associated fungi observed in the plants grown in field soil were the most diverse, whereas the roots isolated from the AMF and autoclaved soil groups presented a similar fungal composition, especially with respect to the abundance of *Fusarium* ssp. (Fig 4). Other studies on fungal communities colonizing wheat plants have indicated that geographical location, cultivar, growth stage, and leaf position in phyllosphere [42], host maturity and host organ in endosphere [40, 41] and management strategies [41] significantly impacted the fungal reservoir in *Triticale*.

The ‘Zollernspelz’ spelt wheat cultivar analyzed in this study is a modern, high-yielding variety cultivated in central Europe [44], and our results showed that this cultivar demonstrated the most unique endophytic pattern, as it had the highest number of exclusive endogenous fungi (Fig 3). However, PCoA analysis did not reveal the significant impact of host genotype in roots mycobiome in studied wheat cultivars. By contrast, previous studies indicated that host genotype affects fungal communities structure in wheat phyllosphere on species level (wheat, barley, oat, rye, triticale), as well as cultivar level (6 wheat cultivars) [45]. Presumably, the impact of host genotype on below ground wheat organs mycobiome is limited, or applied by the authors more sensitive method (culture independent approach) influenced obtained results.

Till date, no study has explored how the structures of fungal communities in wheat and spelt wheat roots are affected by drought stress. However, Vujanovic et al. (2019) [46] recently investigated the impact of drought on *Triticum durum* L. var. durum plants. The authors showed that if the first-generation seeds of *T*. *durum* plants were pretreated with an endophytic plant growth promoter (*Penicillium* sp. SMCD 2318), the second- and third-generation plants exhibited higher drought resistance and positive phenotypic changes under drought stress. We did not observe a significant impact of drought on the composition of fungal communities in wheat roots. Obtained results are in agreement with the recent work, were the abundance of microorganisms residing in rhizosphere of non-irrigated and irrigated wheat plants, were measured using real-time PCR [47]. The authors did not observe significant changes in fungal ITS region abundance in studies samples. Interestingly, the bacterial 16S gene copies were less abundant in non-irrigated soil. Obtained result suggest that fungi are more resistant on water changes in the soil, than bacterial communities. Hawkes et al. (2011) [48] observed greater fungal diversity and abundance in soil with periodically low rainfall and assumed that drought stress moderates competition between fungi. However, we identified similar number of different taxa in rhizosphere under drought and controlled condition (16 and 17, respectively; Table 2) Probably, the composition of fungi in soil closely surrounding roots and from adjacent area presenting different relations under water deficiency conditions. Moreover, we did not notice any drought-specific pattern of fungal composition in the roots of the studied plants, however the *M*. *hedericola*, *A*. *verrucaria*, *G*. *radicicola*, and *A*. *salicis* and the species from *Zopfiella* genus, observed exclusively in the irrigated group, as well as *T*. *longibrachiatum* and *T*. *velutinum*, found only in the plants grown under drought stress. It is worth mentioning that *Trichoderma* sp. are known to be associated with plant roots, where these fungi either form a symbiotic relationship or occur as plant endophytes [49–51]. Studies have shown that *Trichoderma* strains exerted direct effects on host plants by increasing their growth potential and nutrient uptake, efficiency of fertilizer use, percentage and rate of seed germination, and their ability to withstand abiotic stresses, such as drought, salinity, and high temperature, as well as biotic stresses by stimulating their defense [50, 52, 53]. In addition, their natural ability to attack other fungi, and in particular their antagonistic activity toward plant pathogens, such as *Botrytis cinerea*, *Fusarium* spp., *Pythium* spp., *R*. *solani*, *Verticillium dahilae*, and *Sclerotinia* spp. [49, 54] has contributed to the recognition of *Trichoderma* spp. as a significant Microbial Biological Control Agent that can help contain the pathogen populations under different agricultural conditions, protect host plants, and enhance vegetative growth, while acting as soil amendments improving the nutrient ability and rate of decomposition and biodegradation [50, 55]. Therefore, the detection of *Trichoderma* spp. in the roots of wheat plants grown under drought stress conditions reported in the present study may indicate their potential to enhance the resistance of host to abiotic stress, which can contribute to improving wheat cultivation in unfavorable conditions. However, to confirm the prevalence of *Trichoderma* spp. in wheat roots under water-deficient conditions, the use of high-throughput technologies (next-generation sequencing of the ITS region) on a larger plant group is recommended. Moreover, to prove the beneficial effects of the isolated *Trichoderma* spp. in terms of increased growth and yield of wheat plants and their enhanced resistance to biotic/abiotic stresses, more comprehensive further research is needed.

Furthermore, our study revealed the coexistence of plant fungal pathogens, symbionts, and commensals in the complex ecosystem of common wheat and spelt wheat roots. Regardless of the growth conditions applied, the genus that was most frequently isolated was *Fusarium*. Nonetheless, under drought conditions, the abundance of *Fusarium* sp. in the wheat rhizosphere was lower in the plants grown in field soil. Presumably, the simultaneous occurrence of antagonistic *Trichoderma* spp. [54] might have caused the observed reduction. *Fusarium* spp., especially *F*. *graminearum*, *F*. *culmorum*, *F*. *avenaceum*, *F*. *poae*, and *F*. *triticum*, are mostly known as causal agents of *Fusarium* head blight, as well as *Fusarium* foot and root rot, and also cause other detrimental changes in wheat plants [56, 57]. A distinct pattern of *Fusarium* colonization was noticed between the rhizosphere and endosphere in the studied wheat groups. Among the irrigated plants, the roots growing in the soil with AMF addition and in the nonsterilized field soil demonstrated a lower amount of *Fusarium* spp. in the endosphere than in the rhizosphere. By contrast, the autoclaved soil group demonstrated a similar abundance of *Fusarium* spp. in both root parts. On the other hand, the situation was diverse under drought conditions; the roots growing in AMF and autoclaved soils showed a reduction of *Fusarium* spp. in the endosphere, whereas in field soil the *Fusarium* colonization was observed to be high in root endosphere. Bokati et al. (2016) [58] observed that *Fusarium* spp. occurred more frequently in root endosphere from desert soil than in clay soil (42% and 23%, when evaluated using a culture-dependent method; 65% and 17%, when culture-independent methods were applied, respectively).

Among the endogenous and epiphytic fungi identified in the study, we observed a large group of microorganisms that have been documented with a positive impact on their host. As mentioned earlier, this group includes species from *Trichoderma* genus (*T*. *ghanense*, *T*. *longibrachiatum*, *T*. *velutinum*, and some unrecognized *Trichoderma* sp.) which have the ability to antagonize plant-pathogenic fungi and stimulate the growth and defense of host plants [51, 54]. In this study, we also identified *A*. *verrucaria* in the rhizosphere of AMF and autoclaved soil. This species was previously isolated from grapes and was also shown to inhibit the growth of *B*. *cinerea* causing green mold on this fruit [59]. However, *A*. *verrucaria* causes stem necrosis and leaf spot in tomato [60]. *Waitea circinata* is an orchid myccorhizal fungus and inhibits the growth of *Magnaporthe oryzae* causing rice blast [61]. Several of the root-associated fungi identified by us belong to the group of latent pathogens. Some of them are major wheat pathogens (e.g. *F*. *poae*, *F*. *culmorum*, and *F*. *avenaceum*), while some identified fungi are known to be pathogens of plants other than wheat; for example, *S*. *terrestris* causes pink root rot in squash, canola, and onion [62, 63]. Moreover, plenty of species with unrecognized impact on wheat plants were identified in this study. The relationship between the identified fungi and wheat plants is unknown, and therefore, additional studies, especially focusing on naturally occurring endogenous fungi that are potential sources of biocontrol agents, are needed.

## Conclusion

A total of 117 fungal strains, belonging to 22 genera, colonizing the internal and external root parts of *T*. *aestivum* ssp. *spelta* L. and *T*. *aestivum* ssp. *vulgare* L. cultivars were isolated. Here, we found that the type of the root part and soil preparation influence the mycobiome composition of the common and spelt wheat roots. Moreover, *Trichoderma longibrachiatum* and *Trichoderma velutinum*, were found exclusively in the plants grown under drought stress. However, water deficiency conditions don’t have significant impact on the roots fungal communities in wheat. In addition, for the first time the fungal reservoir in the root endosphere and rhizosphere of *T*. *aestivum* ssp. *spelta* L. was examined.

## Supporting information

S1 Table NCBI GenBank accession numbers of deposited sequences contained fragments of ITS, SSU and LSU regions. (XLSX)
